# (*E*)-Methyl *N*′-(3,4-dimethoxy­benzyl­idene)hydrazinecarboxyl­ate

**DOI:** 10.1107/S1600536808034260

**Published:** 2008-10-25

**Authors:** Lu-Ping Lv, Xiao-Min Ding, Yong-Zhao Zhang, Wei-Wei Li, Xian-Chao Hu

**Affiliations:** a Department of Chemical Engineering, Hangzhou Vocational and Technical College, Hangzhou 310018, People’s Republic of China; b Research Center of Analysis and Measurement, Zhejiang University of Technology, Hangzhou 310014, People’s Republic of China

## Abstract

The title compound, C_11_H_14_N_2_O_4_, crystallizes with two independent but essentially identical mol­ecules in the asymmetric unit. Each mol­ecule adopts a *trans* configuration with respect to the C=N bond. Mol­ecules are linked into a one-dimensional network by inter- and intra­molecular N—H⋯O and C—H⋯O hydrogen bonds.

## Related literature

For general background, see: Parashar *et al.* (1988 ▶); Hadjoudis *et al.* (1987 ▶); Borg *et al.* (1999 ▶). For a related structure, see: Shang *et al.* (2007 ▶).
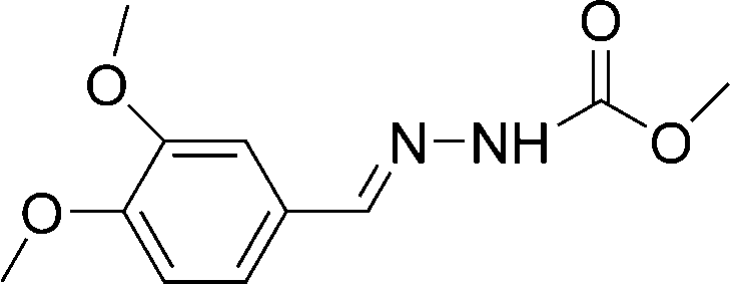


## Experimental

### 

#### Crystal data

C_11_H_14_N_2_O_4_
*M* *_r_* = 238.24
Triclinic, 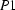

*a* = 8.5276 (11) Å
*b* = 8.5517 (11) Å
*c* = 8.6259 (11) Å
α = 92.919 (5)°
β = 94.209 (4)°
γ = 94.146 (5)°
*V* = 624.71 (14) Å^3^
*Z* = 2
Mo *K*α radiation
μ = 0.10 mm^−1^
*T* = 273 (2) K
0.23 × 0.21 × 0.20 mm

#### Data collection

Bruker SMART CCD area-detector diffractometer
Absorption correction: multi-scan (*SADABS*; Bruker, 2002 ▶) *T*_min_ = 0.971, *T*_max_ = 0.979
3471 measured reflections
2172 independent reflections
1985 reflections with *I* > 2σ(*I*)
*R*_int_ = 0.013

#### Refinement

*R*[*F* ^2^ > 2σ(*F* ^2^)] = 0.028
*wR*(*F* ^2^) = 0.080
*S* = 1.04
2172 reflections
314 parameters
3 restraints
H-atom parameters constrained
Δρ_max_ = 0.11 e Å^−3^
Δρ_min_ = −0.10 e Å^−3^

### 

Data collection: *SMART* (Bruker, 2002 ▶); cell refinement: *SAINT* (Bruker, 2002 ▶); data reduction: *SAINT*; program(s) used to solve structure: *SHELXS97* (Sheldrick, 2008 ▶); program(s) used to refine structure: *SHELXL97* (Sheldrick, 2008 ▶); molecular graphics: *SHELXTL* (Sheldrick, 2008 ▶); software used to prepare material for publication: *SHELXTL*.

## Supplementary Material

Crystal structure: contains datablocks I, global. DOI: 10.1107/S1600536808034260/bg2215sup1.cif

Structure factors: contains datablocks I. DOI: 10.1107/S1600536808034260/bg2215Isup2.hkl

Additional supplementary materials:  crystallographic information; 3D view; checkCIF report

## Figures and Tables

**Table 1:** Hydrogen-bond geometry (Å, °)

*D*—H⋯*A*	*D*—H	H⋯*A*	*D*⋯*A*	*D*—H⋯*A*
N2—H2⋯O5^i^	0.86	2.07	2.902 (3)	164
N2—H2⋯O6^i^	0.86	2.54	3.153 (3)	129
N4—H4*A*⋯O3	0.86	2.13	2.968 (3)	164
C19—H19⋯O2^ii^	0.93	2.55	3.337 (3)	143
C13—H13*A*⋯*Cg*1^iii^	0.96	2.94	3.531 (4)	121

Symmetry codes: (i) 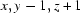; (ii) 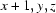; (iii) 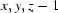. *Cg*1 is the centroid of the C3–C8 ring.

**Fig. 1.**
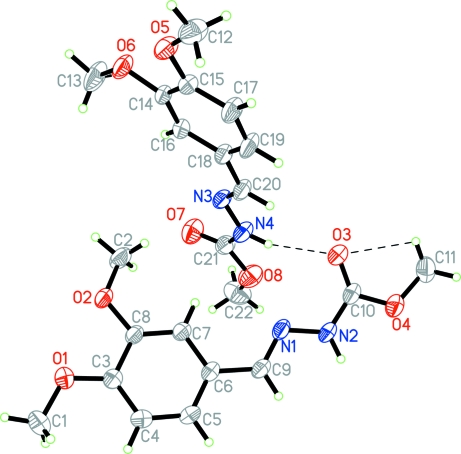
Molecular structure of (I), showing 20% probability displacement ellipsoids and the atomic numbering.

**Fig. 2.**
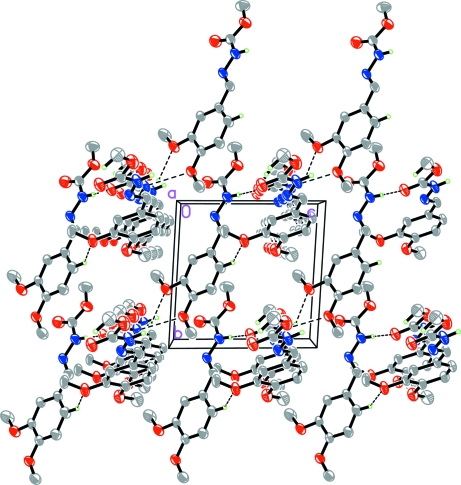
Crystal packing of the title compound, viewed approximately down the a axis. Dashed lines indicate hydrogen bonds. H atoms not intervening in H-bonding were eliminated for clarity.

## supplementary crystallographic information

### Comment

Benzaldehydehydrazone derivatives have received considerable attentions for a long time due to their pharmacological activity (Parashar *et al.*, 1988) and their photochromic properties(Hadjoudis *et al.*, 1987). Meanwhile, it's an important intermidiate of 1,3,4-oxadiazoles, which have been reported to be versatile compounds with many properties(Borg *et al.*, 1999). As a further investigation of this type of derivatives, we report herein the crystal structure of the title compound (I).

The title compound, C_11_H_14_N_2_O_4_ ,crystallizes with two independent, but essentially identical molecules in the asymmetric unit. Each essentially planar molecule of the unit adopts a trans configuration with respect to the C═N bond. in a molecule of the unit,the hydrazine carboxylic acid methyl ester group is slightly twisted away from the attached ring. The dihedral angle between the two essentially planar molecule of the unit is 81.67 (4)°. The bond lengths and angles agree with those observed for (E)-Methyl N'-(4-hydroxybenzylidene)hydrazinecarboxylate (Shang *et al.*, 2007).

The molecules are linked into a one-dimensional network by intermolecular intramolecular N–H···O, C–H···O hydrogen bonds (Fig.2). Meanwhile, A C—H···π contact between benzene ring (centroid Cg1) and H atom of methoxy C13 further stabilizes the structure (Table 1).

### Experimental

3,4-Dimethoxybenzaldehyde (1.66 g, 0.01 mol) and methyl hydrazinecarboxylate (0.9g, 0.01mol) were dissolved in stirred methanol (25ml) and left for 3.2h at room temperature. The resulting solid was filtered off and recrystallized from ethanol to give the title compound in 86% yield. Crystals suitable for X-ray analysis were obtained by slow evaporation of a ethanol solution at room temperature (m.p. 468-470 K).

### Refinement

H atoms were included in the riding model approximation with N-H = 0.86Å. C-bound H atoms were positioned geometrically (C-H = 0.93Å and 0.96Å) and refined using a riding model, with U_iso_(H) = 1.2-1.5U_eq_(C). In the absence of significant anomalous dispersion effects, Friedel pairs were averaged.

### Crystal data

**Table:**

C_11_H_14_N_2_O_4_	*Z* = 2
*M**_r_* = 238.24	*F*(000) = 252
Triclinic, *P*1	*D*_x_ = 1.267 Mg m^−^^3^
Hall symbol: P 1	Mo *K*α radiation, λ = 0.71073 Å
*a* = 8.5276 (11) Å	Cell parameters from 2172 reflections
*b* = 8.5517 (11) Å	θ = 2.4–25.0°
*c* = 8.6259 (11) Å	µ = 0.10 mm^−^^1^
α = 92.919 (5)°	*T* = 273 K
β = 94.209 (4)°	Block, colourless
γ = 94.146 (5)°	0.23 × 0.21 × 0.20 mm
*V* = 624.71 (14) Å^3^	

### Data collection

**Table:**

Bruker SMART CCD area-detector diffractometer	2172 independent reflections
Radiation source: fine-focus sealed tube	1985 reflections with *I* > 2σ(*I*)
graphite	*R*_int_ = 0.013
φ and ω scans	θ_max_ = 25.0°, θ_min_ = 2.4°
Absorption correction: multi-scan (SADABS; Bruker, 2002)	*h* = −9→10
*T*_min_ = 0.971, *T*_max_ = 0.979	*k* = −10→9
3471 measured reflections	*l* = −10→10

### Refinement

**Table:**

Refinement on *F*^2^	Secondary atom site location: difference Fourier map
Least-squares matrix: full	Hydrogen site location: inferred from neighbouring sites
*R*[*F*^2^ > 2σ(*F*^2^)] = 0.028	H-atom parameters constrained
*wR*(*F*^2^) = 0.080	*w* = 1/[σ^2^(*F*_o_^2^) + (0.049*P*)^2^ + 0.0326*P*] where *P* = (*F*_o_^2^ + 2*F*_c_^2^)/3
*S* = 1.04	(Δ/σ)_max_ = 0.010
2172 reflections	Δρ_max_ = 0.11 e Å^−^^3^
314 parameters	Δρ_min_ = −0.10 e Å^−^^3^
3 restraints	Extinction correction: SHELXL97 (Sheldrick, 2008), Fc^*^=kFc[1+0.001xFc^2^λ^3^/sin(2θ)]^-1/4^
Primary atom site location: structure-invariant direct methods	Extinction coefficient: 0.034 (6)

### Special details

**Table:**

Geometry. All esds (except the esd in the dihedral angle between two l.s. planes) are estimated using the full covariance matrix. The cell esds are taken into account individually in the estimation of esds in distances, angles and torsion angles; correlations between esds in cell parameters are only used when they are defined by crystal symmetry. An approximate (isotropic) treatment of cell esds is used for estimating esds involving l.s. planes.
Refinement. Refinement of F^2^ against ALL reflections. The weighted R-factor wR and goodness of fit S are based on F^2^, conventional R-factors R are based on F, with F set to zero for negative F^2^. The threshold expression of F^2^ > 2sigma(F^2^) is used only for calculating R-factors(gt) etc. and is not relevant to the choice of reflections for refinement. R-factors based on F^2^ are statistically about twice as large as those based on F, and R- factors based on ALL data will be even larger.

### Fractional atomic coordinates and isotropic or equivalent isotropic displacement parameters (Å^2^)

**Table:**

	*x*	*y*	*z*	*U*_iso_*/*U*_eq_	
C1	−0.1778 (4)	0.3653 (5)	0.7975 (4)	0.0950 (11)	
H1A	−0.2261	0.2682	0.8289	0.143*	
H1B	−0.2577	0.4272	0.7539	0.143*	
H1C	−0.1225	0.4220	0.8864	0.143*	
C2	0.1762 (4)	0.2088 (4)	0.3329 (3)	0.0765 (8)	
H2A	0.2870	0.2400	0.3409	0.115*	
H2B	0.1272	0.2484	0.2407	0.115*	
H2C	0.1609	0.0962	0.3274	0.115*	
C3	0.0551 (3)	0.2502 (3)	0.7257 (3)	0.0563 (6)	
C4	0.0913 (3)	0.1979 (3)	0.8721 (3)	0.0629 (6)	
H4	0.0267	0.2187	0.9518	0.075*	
C5	0.2224 (3)	0.1151 (3)	0.9009 (3)	0.0614 (6)	
H5	0.2446	0.0798	0.9998	0.074*	
C6	0.3208 (3)	0.0839 (3)	0.7857 (2)	0.0536 (5)	
C7	0.2846 (3)	0.1357 (3)	0.6355 (2)	0.0532 (5)	
H7	0.3497	0.1150	0.5562	0.064*	
C8	0.1542 (3)	0.2161 (3)	0.6063 (2)	0.0518 (5)	
C9	0.4603 (3)	−0.0029 (3)	0.8201 (3)	0.0592 (6)	
H9	0.4750	−0.0452	0.9168	0.071*	
C10	0.8018 (3)	−0.1300 (3)	0.6745 (3)	0.0592 (6)	
C11	1.0411 (4)	−0.2537 (4)	0.6641 (4)	0.0831 (8)	
H11A	1.0612	−0.1752	0.5909	0.125*	
H11B	1.0213	−0.3547	0.6096	0.125*	
H11C	1.1313	−0.2552	0.7374	0.125*	
C12	0.8912 (4)	0.8997 (4)	0.1431 (4)	0.0832 (9)	
H12A	0.8874	0.9151	0.2538	0.125*	
H12B	0.8869	0.9989	0.0963	0.125*	
H12C	0.9876	0.8546	0.1209	0.125*	
C13	0.4029 (4)	0.5264 (5)	−0.1260 (4)	0.1025 (12)	
H13A	0.4310	0.4251	−0.1626	0.154*	
H13B	0.3617	0.5814	−0.2124	0.154*	
H13C	0.3243	0.5137	−0.0525	0.154*	
C14	0.6174 (3)	0.5505 (3)	0.0681 (2)	0.0507 (5)	
C15	0.7418 (3)	0.6523 (3)	0.1397 (2)	0.0537 (5)	
C16	0.5874 (3)	0.4031 (3)	0.1205 (3)	0.0526 (5)	
H16	0.5047	0.3365	0.0728	0.063*	
C17	0.8352 (3)	0.6017 (3)	0.2610 (3)	0.0702 (7)	
H17	0.9194	0.6672	0.3073	0.084*	
C18	0.6816 (3)	0.3529 (3)	0.2460 (3)	0.0567 (6)	
C19	0.8042 (3)	0.4530 (3)	0.3147 (3)	0.0718 (7)	
H19	0.8669	0.4205	0.3980	0.086*	
C20	0.6530 (3)	0.1995 (3)	0.3102 (3)	0.0615 (6)	
H20	0.7189	0.1738	0.3944	0.074*	
C21	0.4159 (3)	−0.1482 (3)	0.2941 (3)	0.0641 (6)	
C22	0.3184 (5)	−0.3964 (4)	0.3679 (5)	0.1022 (11)	
H22A	0.3118	−0.4332	0.2604	0.153*	
H22B	0.2171	−0.3659	0.3945	0.153*	
H22C	0.3501	−0.4787	0.4321	0.153*	
O1	−0.0693 (2)	0.3330 (2)	0.6835 (2)	0.0744 (5)	
O2	0.1079 (2)	0.2698 (2)	0.46445 (18)	0.0699 (5)	
O3	0.8131 (2)	−0.0794 (2)	0.5477 (2)	0.0717 (5)	
O4	0.9058 (2)	−0.2180 (3)	0.7456 (2)	0.0807 (6)	
O5	0.7603 (2)	0.7965 (2)	0.08141 (19)	0.0666 (5)	
O6	0.5370 (2)	0.6126 (2)	−0.0536 (2)	0.0718 (5)	
O7	0.3145 (3)	−0.1471 (3)	0.1902 (3)	0.0887 (6)	
O8	0.4310 (3)	−0.2649 (2)	0.3923 (3)	0.0874 (6)	
N1	0.5617 (2)	−0.0216 (2)	0.7210 (2)	0.0583 (5)	
N2	0.6856 (3)	−0.1066 (3)	0.7687 (2)	0.0703 (6)	
H2	0.6889	−0.1449	0.8591	0.084*	
N3	0.5424 (3)	0.0995 (2)	0.2565 (2)	0.0588 (5)	
N4	0.5318 (3)	−0.0368 (3)	0.3347 (2)	0.0673 (6)	
H4A	0.6002	−0.0504	0.4102	0.081*	

### Atomic displacement parameters (Å^2^)

**Table:**

	*U*^11^	*U*^22^	*U*^33^	*U*^12^	*U*^13^	*U*^23^
C1	0.081 (2)	0.116 (3)	0.097 (2)	0.042 (2)	0.0291 (18)	0.017 (2)
C2	0.099 (2)	0.0901 (19)	0.0422 (12)	0.0300 (16)	−0.0048 (12)	0.0068 (12)
C3	0.0583 (14)	0.0597 (13)	0.0518 (12)	0.0102 (11)	0.0027 (10)	0.0076 (10)
C4	0.0675 (15)	0.0741 (15)	0.0496 (12)	0.0091 (13)	0.0129 (11)	0.0103 (11)
C5	0.0705 (15)	0.0721 (15)	0.0427 (11)	0.0089 (13)	0.0001 (11)	0.0165 (11)
C6	0.0577 (13)	0.0558 (12)	0.0471 (11)	0.0071 (11)	−0.0046 (10)	0.0112 (9)
C7	0.0587 (13)	0.0585 (13)	0.0436 (11)	0.0117 (11)	0.0016 (9)	0.0081 (9)
C8	0.0587 (13)	0.0552 (13)	0.0422 (10)	0.0105 (11)	−0.0015 (10)	0.0090 (9)
C9	0.0674 (15)	0.0662 (14)	0.0452 (11)	0.0133 (12)	−0.0054 (11)	0.0177 (10)
C10	0.0615 (14)	0.0592 (13)	0.0566 (13)	0.0099 (11)	−0.0093 (11)	0.0135 (10)
C11	0.0677 (17)	0.092 (2)	0.090 (2)	0.0219 (15)	−0.0067 (15)	0.0067 (16)
C12	0.091 (2)	0.0815 (19)	0.0730 (17)	−0.0176 (17)	−0.0081 (15)	0.0207 (14)
C13	0.089 (2)	0.105 (2)	0.106 (2)	−0.0075 (19)	−0.053 (2)	0.038 (2)
C14	0.0481 (12)	0.0640 (14)	0.0422 (11)	0.0168 (11)	−0.0008 (9)	0.0126 (10)
C15	0.0551 (13)	0.0624 (14)	0.0448 (11)	0.0102 (11)	0.0017 (10)	0.0110 (10)
C16	0.0486 (12)	0.0607 (13)	0.0496 (11)	0.0114 (10)	−0.0014 (9)	0.0102 (10)
C17	0.0685 (16)	0.0735 (17)	0.0653 (15)	0.0014 (13)	−0.0223 (13)	0.0166 (13)
C18	0.0585 (13)	0.0636 (14)	0.0504 (12)	0.0180 (11)	−0.0002 (10)	0.0132 (10)
C19	0.0718 (17)	0.0781 (18)	0.0643 (15)	0.0126 (14)	−0.0234 (13)	0.0233 (13)
C20	0.0662 (15)	0.0662 (15)	0.0537 (12)	0.0176 (13)	−0.0069 (11)	0.0184 (11)
C21	0.0727 (16)	0.0660 (16)	0.0561 (13)	0.0164 (14)	0.0075 (12)	0.0091 (11)
C22	0.111 (3)	0.071 (2)	0.128 (3)	−0.0024 (19)	0.041 (2)	0.0078 (19)
N1	0.0612 (12)	0.0607 (11)	0.0540 (11)	0.0133 (9)	−0.0076 (10)	0.0181 (8)
N2	0.0719 (13)	0.0897 (16)	0.0546 (11)	0.0293 (12)	−0.0013 (10)	0.0319 (10)
N3	0.0676 (13)	0.0635 (12)	0.0486 (10)	0.0196 (11)	0.0029 (9)	0.0168 (9)
N4	0.0769 (14)	0.0669 (13)	0.0583 (11)	0.0081 (11)	−0.0087 (10)	0.0205 (10)
O1	0.0711 (11)	0.0905 (13)	0.0677 (11)	0.0342 (10)	0.0122 (9)	0.0139 (10)
O2	0.0782 (11)	0.0920 (12)	0.0454 (8)	0.0393 (10)	0.0027 (8)	0.0183 (8)
O3	0.0715 (12)	0.0859 (12)	0.0610 (10)	0.0180 (10)	0.0011 (8)	0.0244 (9)
O4	0.0763 (13)	0.0995 (14)	0.0718 (12)	0.0382 (11)	0.0003 (10)	0.0232 (10)
O5	0.0733 (11)	0.0656 (11)	0.0592 (9)	−0.0022 (9)	−0.0081 (8)	0.0193 (8)
O6	0.0687 (11)	0.0774 (12)	0.0671 (10)	0.0021 (9)	−0.0223 (9)	0.0287 (9)
O7	0.0872 (14)	0.0865 (14)	0.0891 (14)	0.0035 (11)	−0.0180 (12)	0.0127 (11)
O8	0.1069 (16)	0.0683 (12)	0.0869 (13)	0.0002 (11)	0.0003 (12)	0.0223 (10)

### Geometric parameters (Å, °)

**Table:**

C1—O1	1.429 (3)	C12—H12B	0.9600
C1—H1A	0.9600	C12—H12C	0.9600
C1—H1B	0.9600	C13—O6	1.402 (4)
C1—H1C	0.9600	C13—H13A	0.9600
C2—O2	1.407 (3)	C13—H13B	0.9600
C2—H2A	0.9600	C13—H13C	0.9600
C2—H2B	0.9600	C14—O6	1.363 (3)
C2—H2C	0.9600	C15—O5	1.359 (3)
C3—O1	1.355 (3)	C15—C17	1.375 (3)
C3—C4	1.382 (3)	C15—C14	1.407 (3)
C4—H4	0.9300	C16—C14	1.374 (3)
C5—C4	1.381 (4)	C16—H16	0.9300
C5—H5	0.9300	C17—H17	0.9300
C6—C5	1.375 (3)	C18—C19	1.380 (4)
C6—C7	1.411 (3)	C18—C16	1.404 (3)
C6—C9	1.467 (3)	C18—C20	1.461 (3)
C7—H7	0.9300	C19—C17	1.389 (4)
C8—C7	1.363 (3)	C19—H19	0.9300
C8—O2	1.369 (3)	C20—N3	1.271 (3)
C8—C3	1.412 (3)	C20—H20	0.9300
C9—N1	1.272 (3)	C21—O7	1.200 (3)
C9—H9	0.9300	C21—N4	1.336 (4)
C10—O3	1.204 (3)	C21—O8	1.349 (3)
C10—O4	1.339 (3)	C22—O8	1.422 (4)
C10—N2	1.347 (3)	C22—H22A	0.9600
C11—O4	1.438 (4)	C22—H22B	0.9600
C11—H11A	0.9600	C22—H22C	0.9600
C11—H11B	0.9600	N1—N2	1.375 (3)
C11—H11C	0.9600	N2—H2	0.8600
C12—O5	1.425 (3)	N3—N4	1.377 (3)
C12—H12A	0.9600	N4—H4A	0.8600
			
O1—C1—H1A	109.5	O6—C13—H13B	109.5
O1—C1—H1B	109.5	H13A—C13—H13B	109.5
H1A—C1—H1B	109.5	O6—C13—H13C	109.5
O1—C1—H1C	109.5	H13A—C13—H13C	109.5
H1A—C1—H1C	109.5	H13B—C13—H13C	109.5
H1B—C1—H1C	109.5	O6—C14—C16	125.9 (2)
O2—C2—H2A	109.5	O6—C14—C15	113.64 (19)
O2—C2—H2B	109.5	C16—C14—C15	120.47 (19)
H2A—C2—H2B	109.5	O5—C15—C17	124.6 (2)
O2—C2—H2C	109.5	O5—C15—C14	116.08 (18)
H2A—C2—H2C	109.5	C17—C15—C14	119.4 (2)
H2B—C2—H2C	109.5	C14—C16—C18	119.9 (2)
O1—C3—C4	126.0 (2)	C14—C16—H16	120.0
O1—C3—C8	115.39 (19)	C18—C16—H16	120.0
C4—C3—C8	118.6 (2)	C15—C17—C19	120.2 (2)
C5—C4—C3	120.5 (2)	C15—C17—H17	119.9
C5—C4—H4	119.7	C19—C17—H17	119.9
C3—C4—H4	119.7	C19—C18—C16	119.2 (2)
C6—C5—C4	121.0 (2)	C19—C18—C20	118.1 (2)
C6—C5—H5	119.5	C16—C18—C20	122.6 (2)
C4—C5—H5	119.5	C18—C19—C17	120.9 (2)
C5—C6—C7	119.0 (2)	C18—C19—H19	119.6
C5—C6—C9	119.85 (19)	C17—C19—H19	119.6
C7—C6—C9	121.2 (2)	N3—C20—C18	123.1 (2)
C8—C7—C6	120.1 (2)	N3—C20—H20	118.5
C8—C7—H7	119.9	C18—C20—H20	118.5
C6—C7—H7	119.9	O7—C21—N4	127.2 (3)
C7—C8—O2	124.6 (2)	O7—C21—O8	124.9 (3)
C7—C8—C3	120.68 (19)	N4—C21—O8	107.8 (2)
O2—C8—C3	114.69 (19)	O8—C22—H22A	109.5
N1—C9—C6	121.68 (18)	O8—C22—H22B	109.5
N1—C9—H9	119.2	H22A—C22—H22B	109.5
C6—C9—H9	119.2	O8—C22—H22C	109.5
O3—C10—O4	124.9 (2)	H22A—C22—H22C	109.5
O3—C10—N2	126.3 (2)	H22B—C22—H22C	109.5
O4—C10—N2	108.8 (2)	C9—N1—N2	115.30 (18)
O4—C11—H11A	109.5	C10—N2—N1	120.23 (19)
O4—C11—H11B	109.5	C10—N2—H2	119.9
H11A—C11—H11B	109.5	N1—N2—H2	119.9
O4—C11—H11C	109.5	C20—N3—N4	114.63 (19)
H11A—C11—H11C	109.5	C21—N4—N3	120.4 (2)
H11B—C11—H11C	109.5	C21—N4—H4A	119.8
O5—C12—H12A	109.5	N3—N4—H4A	119.8
O5—C12—H12B	109.5	C3—O1—C1	118.0 (2)
H12A—C12—H12B	109.5	C8—O2—C2	117.77 (18)
O5—C12—H12C	109.5	C10—O4—C11	117.2 (2)
H12A—C12—H12C	109.5	C15—O5—C12	118.1 (2)
H12B—C12—H12C	109.5	C14—O6—C13	118.5 (2)
O6—C13—H13A	109.5	C21—O8—C22	116.6 (3)
			
O2—C8—C3—O1	−1.1 (3)	C17—C15—C14—C16	1.2 (3)
C7—C8—C3—C4	−1.2 (3)	C18—C16—C14—O6	178.9 (2)
O2—C8—C3—C4	178.8 (2)	C18—C16—C14—C15	−0.2 (3)
C7—C8—C3—O1	178.9 (2)	O5—C15—C14—O6	1.9 (3)
C8—C3—C4—C5	0.5 (4)	C17—C15—C14—O6	−178.0 (2)
C6—C5—C4—C3	0.6 (4)	O5—C15—C14—C16	−178.9 (2)
O1—C3—C4—C5	−179.6 (2)	C19—C18—C16—C14	−0.3 (3)
C7—C6—C5—C4	−1.0 (4)	C20—C18—C16—C14	178.1 (2)
C9—C6—C5—C4	179.3 (2)	O5—C15—C17—C19	178.4 (3)
O2—C8—C7—C6	−179.2 (2)	C14—C15—C17—C19	−1.6 (4)
C3—C8—C7—C6	0.8 (3)	C18—C19—C17—C15	1.1 (4)
C5—C6—C7—C8	0.3 (3)	C16—C18—C19—C17	−0.1 (4)
C9—C6—C7—C8	180.0 (2)	C20—C18—C19—C17	−178.6 (3)
C5—C6—C9—N1	−173.5 (2)	C19—C18—C20—N3	179.3 (2)
C7—C6—C9—N1	6.8 (4)	C16—C18—C20—N3	0.8 (4)
C6—C9—N1—N2	−179.4 (2)	C18—C20—N3—N4	−178.5 (2)
O3—C10—N2—N1	2.1 (4)	O7—C21—N4—N3	1.5 (4)
O4—C10—N2—N1	−178.7 (2)	O8—C21—N4—N3	−178.0 (2)
C9—N1—N2—C10	−178.3 (2)	C20—N3—N4—C21	176.9 (2)
C4—C3—O1—C1	−2.6 (4)	C17—C15—O5—C12	4.1 (4)
C8—C3—O1—C1	177.3 (3)	C14—C15—O5—C12	−175.8 (2)
C7—C8—O2—C2	15.2 (4)	C16—C14—O6—C13	5.7 (4)
C3—C8—O2—C2	−164.8 (2)	C15—C14—O6—C13	−175.1 (3)
O3—C10—O4—C11	0.3 (4)	O7—C21—O8—C22	0.8 (4)
N2—C10—O4—C11	−178.9 (2)	N4—C21—O8—C22	−179.6 (3)

### Hydrogen-bond geometry (Å, °)

**Table:**

*D*—H···*A*	*D*—H	H···*A*	*D*···*A*	*D*—H···*A*
N2—H2···O5^i^	0.86	2.07	2.902 (3)	164
N2—H2···O6^i^	0.86	2.54	3.153 (3)	129
N4—H4A···O3	0.86	2.13	2.968 (3)	164
C11—H11A···O3	0.96	2.33	2.701 (4)	102
C19—H19···O2^ii^	0.93	2.55	3.337 (3)	143
C13—H13A···Cg1^iii^	0.96	2.94	3.531 (4)	121

Symmetry codes: (i) *x*, *y*−1, *z*+1; (ii) *x*+1, *y*, *z*; (iii) *x*, *y*, *z*−1.
